# Photodynamic Therapy-Adjunctive Therapy in the Treatment of Prostate Cancer

**DOI:** 10.3390/diagnostics12051113

**Published:** 2022-04-28

**Authors:** Michał Osuchowski, David Aebisher, Dorota Bartusik-Aebisher, Magdalena Krupka-Olek, Klaudia Dynarowicz, Maria Przygoda, Aleksandra Kawczyk-Krupka

**Affiliations:** 1 Medical College of the University of Rzeszów, University of Rzeszów, 35-959 Rzeszów, Poland; michal126@interia.pl; 2 Department of Photomedicine and Physical Chemistry, Medical College of the University of Rzeszów, University of Rzeszów, 35-959 Rzeszów, Poland; daebisher@ur.edu.pl; 3 Department of Biochemistry and General Chemistry, Medical College of the University of Rzeszów, University of Rzeszów, 35-959 Rzeszów, Poland; dbartusikaebisher@ur.edu.pl; 4 Department of Internal Medicine, Angiology and Physical Medicine, Centre for Laser Diagnostics and Therapy, Medical University of Silesia in Katowice, 41-902 Bytom, Poland; magda.krupka94@gmail.com; 5 Center for Innovative Research in Medical and Natural Sciences, Medical College of the University of Rzeszów, University of Rzeszów, 35-310 Rzeszów, Poland; kdynarowicz@ur.edu.pl; 6 Students English Division Science Club, Medical College of the University of Rzeszów, University of Rzeszów, 35-959 Rzeszów, Poland; maria.przygoda@interia.pl

**Keywords:** prostate cancer, photodynamic therapy, adjuvant therapy, in vitro methods

## Abstract

The alarming increase in the number of advanced-stage prostate cancer cases with poor prognosis has led to a search for innovative methods of treatment. In response to the need for implementation of new and innovative methods of cancer tissue therapy, we studied photodynamic action in excised prostate tissue in vitro as a model for photodynamic therapy. To ascertain the effects of photodynamic action in prostate tissue, Rose Bengal (0.01 to 0.05 mM) was used as a photosensitizer in the presence of oxygen and light to generate singlet oxygen in tissues in vitro. Five preset concentrations of Rose Bengal were chosen and injected into prostate tissue samples (60 samples with 12 replications for each RB concentration) that were subsequently exposed to 532 nm light. The effects of irradiation of the Rose Bengal infused tissue samples were determined by histopathological analysis. Histopathological examination of prostate samples subjected to photodynamic action revealed numerous changes in the morphology of the neoplastic cells and the surrounding tissues. We conclude that the morphological changes observed in the prostate cancer tissues were a result of the photogeneration of cytotoxic singlet oxygen. The tissue damage observed post photodynamic action offers an incentive for continued in vitro investigations and future in vivo clinical trials.

## 1. Introduction

Despite decades of research involving prostate cancer treatment, chemotherapeutic approaches that result in long term survivability remain elusive [1]. Prostate cancer is the most common solid tumor in men in developed countries [2,3,4,5,6], and it can remain dormant for many years [7]. The main risk factors are age, race, genetic predisposition, diet, obesity, venereal diseases, physical activity, and smoking [8,9,10,11,12,13,14,15]. Most cases of the disease are asymptomatic, requiring PSA tests and biopsy for diagnosis [9,16,17,18]. Approximately 15% of prostate cancer patients are diagnosed with high-risk disease [19]. The disease usually remains undetected, and it manifests in locally advanced or metastatic stages [20]. Testosterone levels have a large influence on the growth of prostate cancer [21]. Due to the morphology and the location of locally advanced prostate tumors, minimally invasive or non-invasive therapeutic methods are preferable. Although there are several clinically approved photosensitizers for the treatment of prostate cancer, photodynamic therapy (PDT) remains a controversial and an uncommon treatment methodology. Photodynamic diagnostics employing photosensitive and fluorescent probes is more common, and it plays an important role in the accurate diagnosis of various malignant neoplasms, including bladder cancer and prostate cancer [22]. Still, PDT is a promising treatment strategy for focal disease treatment in both primary and post-radiotherapy prostate cancer [23]. Photodynamic therapy is a method of treatment that combines the administration of a tissue based or vascular photosensitizer followed by local visible light irradiation to induce the destruction of diseased tissue [24,25]. The destructive photodynamic effect during PDT occurs by interaction of excited state photosensitizers with oxygen to yield cytotoxic reactive oxygen species (ROS) via radical (Type I) or energy transfer (Type II) processes in tumor tissues [26,27]. Reactive oxygen species such as singlet oxygen (Type II), hydroxyl radical, and peroxy radicals (Type I) may initiate apoptotic or paraptotic processes in cancer cells, induce tissue necrosis, and induce both inflammatory and anti-inflammatory immune responses in the body [28,29,30,31].

### Photodynamic Therapy (PDT)

As previously mentioned, photodynamic therapy is an unconventional method of treatment leading to the death of prostate cancer cells [32], and it can only affect areas that can be sufficiently irradiated with light [33,34]. PDT is a useful form of treatment for many types of cancer, and this method requires the administration of a photosensitizer (which accumulates in tumors or in the tumor vasculature), followed by local activation by visible light that is usually delivered by lasers through various types of fibers and endoscopes [35,36,37,38,39]. Endoscopic laser systems have been reported to increase therapeutic efficacy, for example, in antimicrobial testing. Systems have been developed for inhibiting the colonization of bacteria from the Helicobacter pylori strain by ROS initiated cell membrane disruption [35]. Another example is the use of fiber optic devices for localized delivery of singlet oxygen where photosensitizers are immobilized on Vycor glass fiber tips [36,37]. Another example of PDT supports are carbon dots, which effectively inhibit the growth of cancer cells with simultaneous exposure to laser light [38,39].

**Figure 1:**
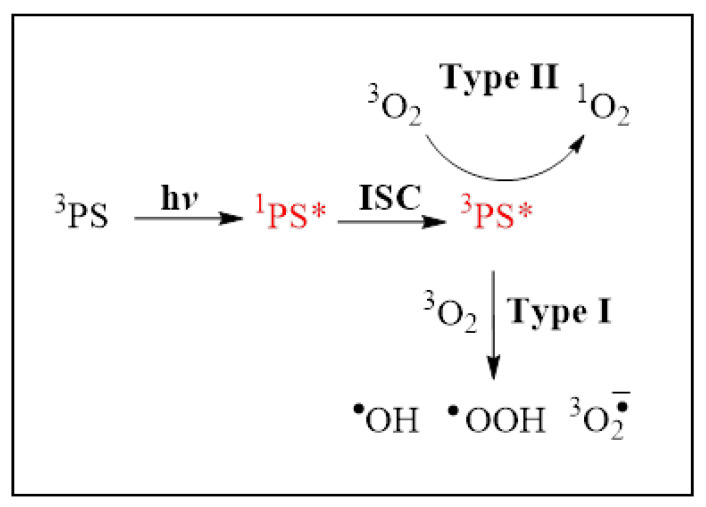
Mechanism of photodynamic ROS generation.

Clinical photodynamic therapy generates reactive oxygen species such as singlet oxygen and peroxy and hydroxyl radicals that damage targeted cancer cells [40]. The mechanism of photodynamic Type I and Type II ROS generation is presented in Figure 1.

Figure 1. As a result of the absorption of light (hv) by the ground state photosensitizer (^3^PS), one electron is transferred to a higher energy orbital to form the excited singlet state (^1^PS*). After intersystem crossing (ISC) to the excited triplet state (^3^PS*) can react in two ways: hydrogen atom abstraction may occur to form radical species (Type I) or the triplet state can react directly with oxygen through energy transfer to form singlet oxygen. Both types of reactions cause cellular cytotoxicity that destroys cancer cells.

Singlet oxygen is capable of oxidizing biological molecules and macromolecules, and it is considered to be the main cytotoxic factor for a majority of clinically approved photosensitizers. Clinical photosensitizers such as Tookad^®^ elicit damage through the formation of hydroxyl radicals. Both Type I and Type II processes can lead to apoptosis [41,42,43].

One photosensitizer commonly used is Rose Bengal. Rose Bengal (4,5,6,7-tetrachloro-2′,4′,5′,7′-tetraiodofluorescein-disodium (RB)) is a hydrophilic anionic photosensitizer that generates singlet oxygen through the Type II pathway [44]. Rose Bengal molecules persist inside cells, localizing in endosomes and then undergoing intracellular redistribution to the perinuclear region and finally to the Golgi apparatus and the endoplasmic reticulum (ER) [45]. Rose Bengal is also effective against various bacterial infections and neoplastic cells [46,47,48]. Rose Bengal is characterized by light absorption between 450 and 600 nm [49], and it has a high singlet oxygen quantum yield [50]. It was used in studies of treatment of larynx cancer [51,52], breast cancer [33], prostatic epithelial cell lines [45], cardiomyocytes [42], nerve axon [42], heart [42], and many others. Despite the progress made in improving PDT, there are limitations due to the limited depth of penetration of light through tissue [52] and local hypoxia of tumors undergoing PDT therapy [53]. Hypoxic neoplastic cells are characterized by significant resistance to PDT compared to neoplastic cells under conditions of constant oxygenation, e.g., in prostate cancer. In vivo studies are significantly challenging and in vitro experiments are most often employed to investigate processes that occur in tissue in order to gain a better understanding of the usefulness of PDT.

In addition, an important element of PDT is the optimization of the distance between the light source and the tissue and the time of exposure to laser light. It is critical to investigate these parameters due to heat generation leading to the drying out of tissues and other morphological changes. In recent years, scientists have been developing optimal tissue irradiation parameters [54,55].

## 2. Materials and Methods

### 2.1. Prostate Tissue Samples

Prostate tissue samples were taken by prostatectomy. Twelve samples were evaluated for each selected concentration of RB. Five selected concentrations of RB were applied to the tissue (0.01–0.05 mM). Prostate cancer control samples were either irradiated without RB (10 samples) or injected with RB without light exposure (10 samples). A total of 80 samples were used. The samples were excised from the peripheral part of the organ (due to the much higher incidence of tumors in this area). The collected samples had a volume of 6 × 4 × 1 mm. The entire sampling process was carried out at the Frederic Chopin Clinical Hospital no. 2 in Rzeszów, Poland. The work presented here is approved by RESOLUTION No. 9/11/2018 of the Bioethics Committee of the University of Rzeszów.

### 2.2. Rose Bengal Concentration

Rose Bengal disodium salt (95%) with a concentration of 0.01 mM (12 samples), 0.02 mM (12 samples), 0.03 mM (12 samples), 0.04 mM (12 samples), and 0.05 mM (12 samples) were applied to prostate tissue samples. Oxygen (99%) gas was purchased from STP & DIN Chemicals, Bielsko-Biała, Poland. The water for the preparation of RB stock solutions was purified with the AquaB Duo reverse osmosis system from Fresenius Medical Care, Singapore. 

**Figure 2:**
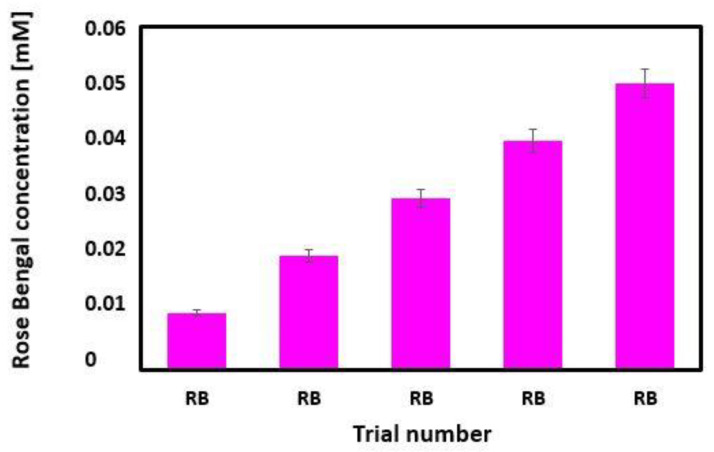
Bar plot of Rose Bengal concentration.

The concentrations of RB used in PDT experiments are presented in Table 1 and Figure 2.

The amount of RB delivered to the tissue varied from 0.01 mM to 0.05 mM. Smaller amounts of the photosensitizer, e.g., 0.01 mM, were very quickly adsorbed. Higher RB values were able to diffuse into the tissue.

### 2.3. PDT Procedure

Samples were injected with various concentrations of RB (0.1 mL) and irradiated at 532 nm for 15 min. The radiant power of the 532 nm light was measured with a Newport power meter model 1918-C. In order to optimize the distance between the laser light source and the prostate tissue, studies were carried out to measure tissue temperature and laser spot size by adjusting the light sequentially 5 cm, 10 cm, and 15 cm from the tissue sample. The effect of irradiation time on Rose Bengal infused tissue was also assessed by microscopic photos at each time point. One hundred cells were counted in the field of view of the microscope after each irradiation time (5 min, 10 min, 15 min). Dead cells were counted for process efficiency, and the percent change was estimated.

**Figure 3:**
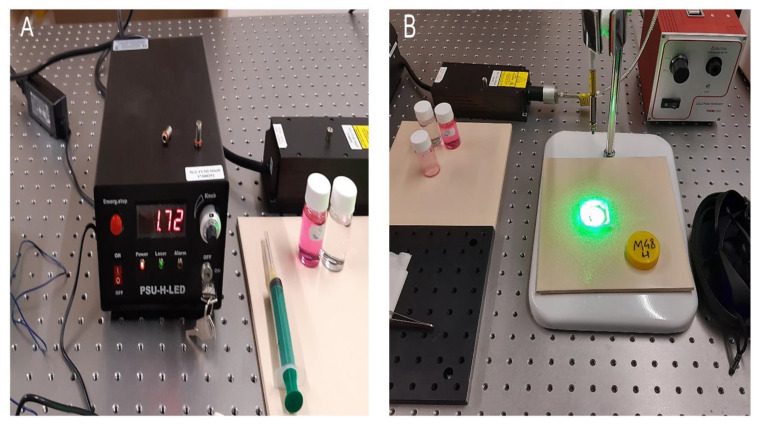
Experimental setup for in vitro irradiation of prostate tissue. Image (**A**) shows an image of the laser power source. Image (**B**) shows irradiation of a prostate cancer tissue sample with laser light.

Figure 3 shows the experimental setup for irradiation of prostate tissue. A solid state laser (LD Pumped All-Solid-State Green Laser, MGL-III-532 nm/300 mW) delivered light with a wavelength of 532 nm, close to the maximum absorption of RB. The laser was connected to a fiber optic cable. The treatment light covered the entire tumor, and it was evenly distributed throughout the tissue. In addition, the distance of the light source from the tissue surface had been selected so as not to cause excessive heating or drying of the tissue. The temperature on the surface of the tissue after 15 min of exposure did not exceed 30 °C. Tissue pieces were cut in half immediately after irradiation. The samples were placed in formalin and subjected to standard histopathological evaluation. Slides fixed and processed in formalin were assessed under an optical microscope. Appropriate controls of photosensitizer without light or light without photosensitizer gave no response.

### 2.4. Histopathological Preparations

The surgical material for histopathological examinations was fixed for 24 h in a 10% buffered formalin solution (4% formaldehyde solution). After fixation of the prostate fragments, tissue sections were collected into cassettes. The tissue material from the cassettes was rinsed, dehydrated, passed through intermediate fluids and embedded in paraffin to obtain blocks. Paraffin blocks were punched on a microtome (Microtom LEICA RM2245) into smaller pieces and placed on slides. Sections were routinely stained with hematoxylin and eosin. For this purpose, a universal device for staining histopathological slides was used (Multistainer LEICA ST 5020). The final step was to cover the sections with a coverslip (LEICA CV 5030 machine) before which the space between the cover glass and the coverslip was filled with histofluid.

### 2.5. Microscope Examination

Histological examinations of the tissues used during the examination were performed at the Clinical Department of Pathomorphology of the teaching hospital of the Provincial Hospital No.1 in Rzeszów. Histological image analysis was performed using a Leica DM1000 LED microscope (LEICA Microsystems, Wetzlar, Germany). Following PDT, cancer cell damage was assessed mainly on the basis of the glandular and the testicular architecture, the degree of chromatin condensation, the presence or the absence of distinct nucleoli, and the severity of stromal swelling. Samples containing benign lesions were assessed similarly.

### 2.6. Statistical Analysis

The data were analyzed using Statistica 13.1 software (StatSoft Polska Sp.z o.o., Krakow, Poland). Values were considered significantly different when the *p*-value was less than 0.05.

## 3. Results

### 3.1. Optimization of PDT Parameters

Changing the light source distance and the exposure time had a significant impact on the efficiency of the in vitro PDT process. The optimization results are presented in Table 2 which shows that the best results were obtained when the prostate tissue was placed 15 cm from the laser light source and exposed to radiation for 15 min. Table 3 shows the results of optimization of the irradiation time of prostate cancer tissue with a laser.

The best PDT efficiency was obtained at a distance of 15 cm from the laser to the sample by irradiating it for 15 min.

### 3.2. Effects on Tissue Morphology of Prostate Cancer after PDT

**Figure 4:**
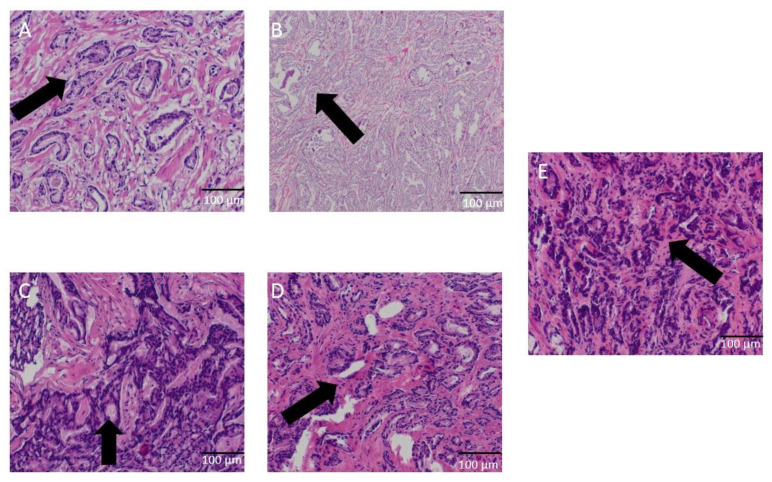
Prostate cancer tissue after PDT with different concentration of RB. Arrows indicate the observed changes presented in the description. Upon application of 0.01 mM RB (**A**), discrete chromatin condensation in most of the cancer cell nuclei and swelling of the stroma are visible. Predominantly normal prostate cancer cells illustrate the irregular arrangement and size of the cells. The architecture of the glands is virtually unchanged, and some cancer cells still have distinct nucleoli. Visible contours of the nuclear membrane. Thrombotic necrosis developed upon application of a concentration of 0.02 mM RB (**B**). In addition to the dense cytoplasmic areas, lighter zones that have a pinkish color after treatment with Rose Bengal were detected. PDT with the application of Rose Bengal at a concentration of 0.03 mM (**C**) showed mild chromatin condensation, irregular shape of the nuclei, and significant architectural disturbances. The cells shown are surrounded by dark cells that stain much more intensely. After the application of 0.04 mM RB (**D**), mild to massive chromatin condensation, pyknotic nuclei, and significant architectural disturbances were observed. These changes were also accompanied by swelling and the presence of protein in the stroma. Fragment of epithelium in a small percentage of cells with the presence of homogeneous chromatin with almost complete loss of chromatin granularity was observed. In the last stage after the application of 0.05 mM RB (**E**), enhanced traits of cell damage and necrosis, which are easily identified. Cell nuclei and whole cells stick together, which makes them indistinguishable. There is also swelling and the presence of protein in the stroma. All microscopic images are at 63× magnification. Histological examination of prostate tumor tissues showed PDT damage to the testicular cells of the tumor cells.

Histologists have been utilizing microscopic techniques to investigate certain aspects of cell structure after PDT. Prostate tissue staining showed that after PDT treatment with Rose Bengal water solution, the number of tumor cells decreased when compared to the number of tumor cells before PDT treatment. After PDT, cell sizes were various, incomplete, and fragmented. Figure 4 shows a microscopic images of prostate tissue after PDT with visible RB staining from a tissue layer not exceeding 2 mm deep. By using increased concentrations of Rose Bengal, cells tend to condense with a uniform nuclear chromatin, fuzzy, no visible nuclei, no visible granularity, and an invisible nuclear membrane. Some cancer cells were unchanged but had various sizes with bright chromatin and an uneven distribution, visible nucleoli, and visible contours of the nuclear membrane. There can also be visible cancer cells with enlarged polymorphs with unevenly distributed translucent chromatin and enlarged irregular nucleoli. Histology images of prostate cancer showed abundant chronic inflammatory infiltration.

Histological images showed visible epithelium of cancer with a picture of cellular pyknosis. In the case of healthy prostate tissues, histological images after PDT differed from those for cancerous tissues. In the case of healthy tissues, areas with damage due to PDT were found much less frequently and in a small area.

As a result of PDT with Rose Bengal at concentrations in the range of 0.01–0.05 mM, similar but not identical changes in tissue morphology were observed under the microscope. The effects of PDT were very significant. The results of PDT on prostate cancer cells in vitro are presented in Table 4.

## 4. Discussion

Prostate cancer statistics, grading, diagnosis and treatment strategies and treatment possibilities and options were often a main subjects of excellent reviews [56,57].

For PDT to be effective, a lot depends on the type of photosensitizer used and its concentration. Based on recent reviews and experiments, PS has been characterized in terms of their effectiveness and selectivity. Several generations of PS used have been distinguished. One of them (also used in our experiment) is RB. PDT induces several cell changes that include the induction of apoptosis, DNA damage, and the induction of an inflammatory response. RB is an efficient producer of singlet oxygen in PDT, and it is also used with chemical modification.

Our application of RB showed that after PDT, the number of tumor cells decreased when compared to the number of tumor cells prior to PDT. After PDT, the cell sizes were various, incomplete, and fragmented.

In our experiment, we performed identification of the histopathological changes caused by PDT with the use of an optical microscope, and we evaluated the effectiveness of damaging prostate cancer cells with the use of RB. Histopathological examination of samples subjected to PDT revealed numerous changes in the morphology of the cancer cells. PDT with RB has shown to be an effective method of damaging prostate cancer cells, and it may prove useful as a method of assessing the effects of PDT in various cancers in vitro. Both the increased RB concentrations and the increased oxygen level contribute to the final efficacy of therapeutic effect.

An example of experimental studies using RB in PDT are studies conducted by Cronin et al. Cronin et al. described the photoactivation of Rose Bengal with a green laser (λ = 532 nm) at fluences of 68, 133, and 228 J/cm^2^ to assess the fungicidal effect on T. rubrum spore suspensions. A 140 µM RB solution was able to induce a fungicidal effect on T. rubrum after photosensitization at a fluidity of 228 J/cm^2^. As a result of the experiment, RB activated with green laser light has become a potentially new treatment for T. rubrum infection [58]. In turn, Uekubo et al. combined RB with a blue LED in Antimicrobial Photodynamic Therapy (a-PDT). Gingival cells were treated with RB (450–470 nm; 1 W/cm^2^, 5 s) or RB + blue light under both anaerobic/aerobic conditions. Under anaerobic conditions, RB + blue light significantly inhibited bacterial growth after 18 h. On the other hand, under aerobic conditions, RB + blue light immediately influenced the growth of bacteria and completely inhibited growth for 48 h. As a result of the experiment, it was concluded that the RB + blue light oxygen treatment may have a bactericidal effect through the a-PDT effect, causing the destruction of RNA and bacterial cells in a short time [59]. In the case of larynx tissues, studies conducted by Bartusik-Aebisher et al. on in vitro applied RB in laryngeal tissues (at a concentration of 0.1, 0.2, 0.3, 0.4, 0.5, 1, and 1.5 mM) caused significant changes in the structure of cells. Among other issues, there was a complete loss of chromatin granularity and the nucleoli became enlarged and irregular [52]. This study also assessed the tissue penetration of the photosensitizer used. PDT induced changes in the morphology of laryngeal cancer cells at a depth of 2–3 mm (2 mm photosensitizer penetration depth, 3 mm laser penetration depth). Above this depth, no changes were observed. This observation indicates some limitations in the use of photosensitizer and laser in in vivo studies due to the low tissue penetration of light.

The effectiveness of PDT based on the analysis of microscopic images was also carried out on breast cancer tissues. Morphological changes of tissues were assessed depending on the concentration of the photosensitizer used. Rose Bengal was also used as a photosensitizer. As the concentration of the photosensitizer increased, chromatin condensation of cancer cells became more and more visible, while at the same time the pyknosis of the cell nuclei was enhanced. The study confirmed the usefulness and the effectiveness of in vitro PDT in the treatment of cancer [60].

Rose Bengal was also used in breast cancer tissue to assess the effectiveness of PDT in a microscopic examination. The degree of changes in neoplastic cells was directly proportional to the concentration of the photosensitizer used. The morphology of the cancer cells was disturbed, the size of the cells was reduced, the cell nuclei were reduced, and they became irregular. The chromatin became lumpy, and it thickened. The conducted study also confirmed the effectiveness of in vitro PDT therapy on breast cancer cells [61]

Sun et al. confirmed the effectiveness of PDT in prostate cancer. The conducted PDT experiment led to apoptosis of prostate cancer cells [62]. In turn, Xu et al. used human prostate cancer cell lines with PDT. Based on the analyzed microscopic results, the photosensitizer used and the exposure of the cells to laser light caused the cancer cells to enter apoptosis. The experiment also analyzed protein extraction and determination. After PDT, a decreased expression of two mitochondrial membrane proteins was found [63].

## 5. Conclusions

Despite advances in optical methods for generating singlet oxygen, PDT remains a challenge for in vivo applications due to the limited depth of light and to photosensitizer penetration through the tissue. Since PDT is a new method of cancer treatment, it may be associated with various new problems, such as a poor accumulation of photosensitizers into the tumor, low penetration of laser light, or microenvironmental hypoxia of the tumor tissue, which limit (to a greater or lesser extent) its therapeutic effectiveness. However, the conducted experiment confirmed the effectiveness of PDT in reducing the number of neoplastic cells, which gives opportunities and hopes for the use of PDT in in vivo clinical trials in patients with prostate cancer.

## Figures and Tables

**Table 1:** Concentration of rose bengal.

Photosensitizer	Rose Bengal Concentration [mM]
Rose Bengal	0.01
	0.02
	0.03
	0.04
	0.05

**Table 2:** Results of optimization of the distance between the laser light source and the sample.

The Distance between the Laser Source and Tissue [cm]	Power of 532 nm Light Dose $\left[\frac{J}{cm^{2}}\right]$	Comments
5	30	If the laser source was 5 cm from the tissue, the sample heats up to 30 °C and the light beam is twice the size of the sample.
10	16	If the laser source was 10 cm from the tissue, the sample heats up to 28 °C and the light beam was bigger than tissue sample diameter.
15	9	If the 532 nm light source was placed at a distance of 15 cm, the light covered the correct area of tissue. The temperature on the surface of the tissue was room temperature and did not dry out the tissue during the course of the experiment. Additionally, conducting the experiment within 15 min turned out to be sufficient for maximum efficiency of PDT in vitro. Extending the time to 20 or 30 min (twice) did not affect the changes visible under the microscope.

**Table 3:** Results of optimization of the irradiation time of prostate cancer tissue with a laser.

Time [min]	Distance [cm]	Percentage of Death Cells [Counted %]
5	15	32±9
10	15	75±9
15	15	100±11

**Table 4:** Microscopic changes after PDT use.

Rose Bengal Concentration [mM]	Cell Changes Percentage (%)	Number of Samples for Testing
0.01	Discreet chromatin condensation and stromal oedema. 45% ± 10% of cells	12
0.02	Thrombotic necrosis. The PDT changes cannot be assessed in this sample. 60% ± 5% of cells	12
0.03	Mild chromatin condensation and irregular shape of nuclei. Stromal oedema. 75% ± 11% of cells	12
0.04	Mild chromatin condensation and irregular shape of nuclei. Significant stromal oedema. 80% ± 5% of cells	12
0.05	Pyknotic nuclei of the glands. Significant oedema. Presence of protein content in the stroma. 100% ± 10% of cells	12

## Data Availability

The data presented in this study are available on request from the corresponding author. The data are not publicly available due to ethical issues.
